# Sterilization and Disinfection: Ensuring Infection Control in Dental Practices

**DOI:** 10.7759/cureus.79041

**Published:** 2025-02-15

**Authors:** Nuria Patiño-Marín, Lorena Dafnee Villa García, Eva Concepción Aguirre López, Carlo Eduardo Medina-Solís, Alan Martínez Zumarán, Ricardo Martínez Rider, Raúl Márquez Preciado, Pedro Rosales García, Marco Felipe Salas Orozco

**Affiliations:** 1 Clinical Research Laboratory, Faculty of Stomatology, Autonomous University of San Luis Potosí, San Luis Potosí, MEX; 2 Dentistry, Health Sciences Institute, Autonomous University of the State of Hidalgo, San Luis Potosí, MEX; 3 Orthodontics and Dentomaxillofacial Orthopedics, Faculty of Stomatology, Autonomous University of San Luis Potosí, San Luis Potosí, MEX; 4 Oral and Maxillofacial Surgery, Faculty of Stomatology, Autonomous University of San Luis Potosí, San Luis Potosí, MEX; 5 Pediatric Dentistry, Faculty of Stomatology, Autonomous University of San Luis Potosí, San Luis Potosí, MEX; 6 Faculty of Stomatology of the Northeastern Regional Complex, Meritorious Autonomous University of Puebla, Puebla, MEX

**Keywords:** autoclave, dentistry, disinfection, infection control, sterilization

## Abstract

Effective sterilization and disinfection are critical for infection control in dental practices, reducing healthcare-associated infections and ensuring patient safety. This review explores the principles, applications, and limitations of various sterilization and disinfection methods used in dentistry, including heat sterilization (steam and dry heat), chemical sterilization (ethylene oxide, hydrogen peroxide), and radiation methods (ultraviolet (UV) and gamma rays). Emphasis is placed on the effectiveness of each method against a range of pathogens, their suitability for different dental instruments, and advancements in technology such as vaporized hydrogen peroxide systems and antimicrobial coatings. Autoclave sterilization remains a cornerstone due to its reliability, while methods like UV rays and ozone offer innovative, material-friendly alternatives. The importance of verifying sterilization efficacy through biological indicators and maintaining proper storage protocols to ensure sterility is also highlighted. By integrating traditional techniques with emerging technologies, dental practices can enhance infection control standards while adapting to modern challenges.

## Introduction and background

Healthcare-associated infections (HAIs) represent a critical concern in clinical settings, including dental and medical practices, where they constitute one of the most common adverse outcomes of patient care. Contaminated surfaces and reusable instruments often act as reservoirs for pathogens, facilitating the transmission of infections. Nosocomial infections, particularly those caused by inadequate sterilization and disinfection, pose significant risks to patient safety and underscore the importance of stringent infection control protocols [1].

The sterilization of medical and dental instruments is vital to prevent cross-contamination and ensure patient safety. This process involves a combination of cleaning, disinfection, and sterilization steps tailored to the nature and intended use of each instrument. Traditional methods such as steam sterilization remain widely utilized for their efficiency in eliminating microorganisms, while alternative approaches like low-temperature chemical sterilization and radiation techniques cater to heat-sensitive materials. Furthermore, the introduction of advanced technologies such as vaporized hydrogen peroxide and ultraviolet irradiation has revolutionized sterilization practices, offering enhanced efficacy and safety [2].

Central to effective sterilization is the integration of maintenance protocols and the use of biological indicators to monitor the sterility assurance level (SAL). These measures ensure the reliability of sterilization cycles and help mitigate risks associated with equipment malfunctions or human errors. In addition, the implementation of proper storage and transport practices plays a critical role in maintaining sterility until the point of use [3].

This review provides a comprehensive analysis of sterilization and disinfection methods, highlighting their principles, applications, and limitations. Emphasis is placed on the integration of innovative technologies and adherence to international guidelines to advance infection control practices in clinical settings.

## Review

Disinfection and washing of dental instruments

HAIs are the most common adverse outcomes due to the delivery of medical care. Contaminated surfaces, particularly those that are touched frequently, act as reservoirs for pathogens and contribute towards pathogen transmission [4]. Each surgical procedure involves the contact of a patient's sterile tissue and/or mucous membrane with a medical device or surgical instrument, posing a major risk of introducing infection [5]. 

Nosocomial infections are a serious problem in healthcare facilities. Bacteria can be transferred from patient to patient via contaminated reusable medical devices and equipment [6]. The clinical environment also represents an essential reservoir for the cross-transmission of healthcare-associated infections and the application of procedures related to handwashing and disinfection and sterilization of surfaces and instruments remain key strategies to control healthcare-associated infections [7]. Disinfection goes with cleaning beforehand to reduce the contamination load and interfering organic matter. Therefore, routine cleaning and disinfection are combined. It comprises the removal of organic soil or dirt using a neutral detergent or microfibre cloth, followed by the application of disinfectant if required. Disinfectants cannot achieve their full potential in the presence of residual surface dirt [4].

Instrument Washing

By definition, cleaning comprises the physical removal of dust and soil using water, with or without detergent and mechanical action, until visibly clean [4].

The central processing areas: Ideally the processing areas should be divided into at least three areas: cleaning, packaging, and sterilization and storage. Physical barriers should separate the cleaning area from the other sections to contain contamination on used items. Occupational exposure limits such as ceiling exposure value (CEV) for chemical agents (e.g. glutaraldehyde, ethylene oxide) are to be complied with in accordance with local environmental law. In healthcare settings where there are dedicated central reprocessing areas, negative pressure airflow must be maintained in cleaning areas and positive pressure airflow must be maintained in clean areas and monitored regularly [8]. Effective cleaning and disinfection practices, such as chemical disinfection, heat, and ultraviolet (UV) germicidal irradiation play a key role in preventing cross-contamination [9]. Medical devices can be cleaned manually or by automated washers. Several studies have documented that the removal of bioburden and organic residues by the cleaning process is more effective and reproducible when automated cleaning machines are used [10-12]. Studies showed that a washer-disinfector was extremely effective in eliminating microorganisms (>7-log10 reduction), including vegetative and spore-forming bacteria, from experimentally contaminated instruments [13].

Instrument Disinfection

The disinfection process aims to eliminate many or all pathogenic microorganisms to reduce the risk of infection and cross-contamination [4]. Medical devices are washed, disinfected, and sterilized before being used for invasive clinical procedures such as surgery, to prevent pathogen transfer [14]. Terminal cleaning and disinfection of an instrument take place after a patient has been discharged to prevent potential pathogens on surfaces from spreading to the next patient to occupy the area [4]. Most medical and surgical devices used in healthcare facilities are made of materials that are heat stable and thus are sterilized by heat, primarily steam sterilization. Since 1950, however, there has been an increase in medical devices and instruments made of materials (eg, plastics) that require low-temperature sterilization. Ethylene oxide (ETO) gas has been used since the 1950s for heat-sensitive and moisture-sensitive medical devices. Within the past 25 years, several new, low-temperature sterilization methods have been developed [15]. The level of disinfection is dependent on the intended use of the object: critical (items that contact sterile tissue such as surgical instruments), semicritical (items that contact mucous membrane), and noncritical (devices that contact only intact skin) items require sterilization, high-level disinfection, and low-level disinfection, respectively. Cleaning must always precede high-level disinfection and sterilization. When properly used, disinfection and sterilization can ensure the safe use of invasive and non-invasive medical devices [5].

Disinfectant as the main constituent for disinfection action has a crucial impact on the decontamination process. The antimicrobial activity of disinfectants performs in two different ways: growth inhibition (e.g. bacteriostatic, fungistatic) and lethal action (sporicidal, bactericidal, fungicidal, and virucidal effects). Disinfectants comprise a wide variety of active chemical agents (biocides) [16]. The active ingredients found in the market are generally alcohols, chlorine, aldehydes, peroxygens, and quaternary ammonium compounds. Every type of disinfectant presents some advantages and disadvantages allowing, or not, its use in wipes [9]. The most commonly used disinfectants were alcohol, ortho-phthalaldehyde (OPA) solution, hydrogen peroxide, peracetic acid, and glutaraldehyde [17]. Alcohol is cheap and easy to obtain allowing an efficient wetting of the surfaces with a rapid bactericidal effect without bacteriostatic action and relevant toxicity issues. However, it is highly inflammable, corrosive to metals, and lacks efficacy in the presence of organic dirt [9].

Hydrogen peroxide presents a satisfying germicidal activity including bacterial spores (with longer contact time). It is relatively environmentally friendly due to its fast degradation. An accelerated hydrogen peroxide (AHP) was specifically developed for widened material compatibility and application variability. It can cause chemical irritation resembling pseudomembranous colitis [18,19].

Quaternary ammonium compounds (QACs) are the most commonly used disinfectants in ordinary environmental surfaces with good cleaning and deodorization properties and most importantly, a broad spectrum of biocidal and sporostatic activity (lipid, enveloped viruses). The incorporation of quaternary ammonium moieties into polymers showed an effective antimicrobial effect against biofilm. Nowadays, QACs are the most used disinfectant in wipes. However, they also have some drawbacks such as a susceptibility to high water hardness and low efficacy against gram-negative bacteria and non-enveloped viruses. Moreover, numerous studies showed that the adsorption of QACs onto the cotton substrate wiping material could lead to the failure of the disinfection process [9].

Glutaraldehyde is a dialdehyde that displays potent bactericidal, fungicidal, mycobactericidal, sporicidal, and virucidal activity. Pertinent to its activity is its interaction with amino groups in proteins and enzymes, but this simplistic statement masks the manner in which it inactivates various types of microorganisms. Notwithstanding its toxicity for medical staff, glutaraldehyde remains an invaluable compound for high-level disinfection purposes [20]. Glutaraldehyde is effective against microorganisms and their spores. Studies have shown the effectiveness of glutaraldehyde against the HIV virus. The use of 2% glutaraldehyde is now recommended for the sterilization of surgical instruments and operating areas [21].

Levels of disinfection

Low Level Disinfection of Noncritical Items

Low-level disinfectants are used for the disinfection of noncritical environmental surfaces and equipment in healthcare facilities. Their use is supported by excellent evidence in the scientific literature that contaminated environmental surfaces and noncritical patient care items play an important role in the transmission of several key healthcare-associated pathogens [22]. A portable device for cleaning and ozone sterilization of small-sized delicate dental instruments that cannot withstand the high heat and humidity of standard autoclaving has been developed. The device contains a remote unit for magnetic mechanical washing, an ultrasonic bath for pre-cleaning treatment, and a container for ozone sterilization with a reactor based on dielectric barrier discharge [23].

High-level Disinfection of Semicritical Items

Semicritical items are those that come in contact with mucous membranes or nonintact skin. Semicritical items minimally require high-level disinfection using chemical disinfectants. Glutaraldehyde, hydrogen peroxide, OPA, peracetic acid, and peracetic acid with hydrogen peroxide, and a chlorine-based system have been cleared by the FDA [18], and are dependable high-level disinfectants provided the factors influencing germicidal procedures [15,24].

High-level Disinfection of Critical Items

Critical items are those that come in contact with sterile tissue such as surgical instruments [5]. Glutaraldehyde is the disinfectant of choice in high-level disinfection of critical surfaces [20]. With the exception of 0.2% peracetic acid (12 minutes at 50-56°C), the indicated exposure times for liquid chemical sterilants range from 3-12 hours. Liquid chemical sterilants can be relied upon to produce sterility only if cleaning, which eliminates organic and inorganic material, precedes treatment and if proper guidelines as to concentration, contact time, temperature, and pH are met [5]. One limitation to the sterilization of devices with liquid chemical sterilants is that the devices cannot be wrapped during processing in a liquid chemical sterilant; thus, it is impossible to maintain sterility following processing and during storage. Furthermore, devices may require rinsing following exposure to the liquid chemical sterilant with water that, in general, is not sterile. Therefore, due to the inherent limitations of using liquid chemical sterilants in a nonautomated (or automated) reprocessor, their use should be restricted to reprocessing critical devices that are heat sensitive and incompatible with other sterilization methods. Cleaning must precede high-level disinfection and sterilization to minimize or eliminate organic and inorganic material as well as microbial load [10,24].

Sterilization methods

There are various methods used for sterilizing instruments in medicine and dentistry, aiming to eliminate or destroy all pathogenic microorganisms, including spores. These methods are fundamental to ensuring the safety of clinical procedures and preventing nosocomial infections [25,26]. The most employed sterilization methods include heat sterilization (dry or moist), chemical sterilization, radiation, and gas sterilization. Each method has specific characteristics that make it more or less suitable depending on the type of material to be sterilized, its nature, and the clinical context. Within heat sterilization, dry heat sterilization and moist heat sterilization are the two most commonly used methods in dentistry [25-27].

Sterilization by Pressurized Steam

Moist heat sterilization is performed using pressurized steam, allowing bacterial death through the coagulation of bacterial cellular proteins. This process is carried out by directly exposing the instruments to a temperature of 121-132°C at 12 psi for a minimum of 15 minutes. The sterilization time may vary depending on the quantity and density of items inside the autoclave chamber, a device widely used in medical and dental fields [27-29].

Among the advantages of this method is its applicability to materials such as cotton, thread, synthetic fibers, liquids like distilled water or solutions that do not alter their composition, glass, rubber, and heat-resistant plastics. This method is non-toxic, has antimicrobial and sporicidal actions, and enables rapid penetration into all types of instruments. However, it also has disadvantages such as the corrosion and even combustion of heat-sensitive materials. Furthermore, it is crucial to ensure that instruments are perfectly clean and free of residues, as this can increase structural damage in materials such as burs and other rotary cutting instruments. An essential aspect to analyze in autoclave equipment and their use is their classification, which varies depending on the method of air extraction from the chamber. These classifications include types N, B, and S. Type N autoclaves are designed for solid, unwrapped instruments intended for immediate use, meaning they cannot be stored for several days after the process. Type B autoclaves are suitable for sterilizing all thermo-stable bagged materials, allowing the sterilized material to be stored for a certain period. On the other hand, type S autoclaves have factory-defined cycles and specific programs for each type of load, depending on its composition [28].

Autoclaves must be checked regularly to ensure that their sterilization process is effective. However, the periodicity of these checks may vary depending on the specific regulations and conditions of each country, as local health authorities may establish different monitoring frequencies based on national guidelines and risk assessments. In this sense, *Geobacillus stearothermophilus *is used as a biological indicator to check the performance of the autoclave. If the indicator shows a color change or turbidity, it indicates spore growth, invalidating the sterilization process; if there are no changes, the process is considered validated. Some biological indicators on the market include a pH indicator, which changes color when spore growth occurs, making it easier to detect [30]. Ardeshna et al. evaluated bacterial contamination on orthodontic appliances and tested various sterilization methods, including steam autoclave. The appliances were contaminated with bacteria such as *Staphylococcus aureus*, *Staphylococcus epidermidis*, *Lactobacilli*, and *Klebsiella pneumoniae*. The autoclave method was highly effective in eliminating all bacterial contamination, providing reliable sterilization [1].

Dry Heat Sterilization

Dry heat sterilization is another common method of sterilization, performed by using hot air generated in an oven designed for this purpose. This process causes the oxidation of microbial proteins, leading to cell death. To achieve this, a temperature of 160°C must be maintained for at least 60 minutes [27]. This method has the advantage of low operational costs and is non-corrosive, unlike autoclaves. Therefore, it is used for sterilizing materials that may be damaged by moist heat such as burs and other rotary instruments. However, this method can degrade the properties and plasticity of many orthodontic materials. Additionally, it cannot be used for materials like plastics, fabrics, or liquids, as it could cause combustión [25,27,31].

Dry heat equipment also requires verification to ensure the effectiveness of its sterilization process. *B. atrophaeus *is the biological indicator that evaluates the functioning of this equipment [32]. Ardeshna et al. investigated bacterial contamination on orthodontic materials such as wires, elastomeric chains, and ties, identifying microorganisms like *S. aureus*, *S. epidermidis*, *Lactobacilli*, *K. pneumoniae*, *Bacillus licheniformis*, and *Bacillus cereus*. Among the sterilization methods tested, dry heat at 121°C for 30 minutes effectively eliminated all bacterial contamination. While effective, the study noted that dry heat sterilization could potentially degrade the properties of some orthodontic materials, making its use dependent on the specific material's tolerance to heat [1].

Chemical Sterilization

Chemical sterilization is crucial for thermosensitive dental instruments or materials that cannot withstand high-temperature methods, such as steam sterilization. Among the most common techniques are the use of unsaturated chemical vapor, 2% glutaraldehyde, and hydrogen peroxide. These chemical agents effectively eliminate pathogenic microorganisms without compromising the integrity of the instruments, ensuring their functionality and safety in medical and surgical procedures [27,33,34].

Unsaturated Chemical Vapor

Unsaturated chemical vapor sterilization, although not one of the most widely used methods, is particularly effective for steel instruments such as dental burs and other rotary instruments. This process utilizes a chemical solution, usually composed of alcohol and formaldehyde, heated to 270°C in a pressurized chamber. Sterilization is achieved within a time range of 20-40 minutes. Its primary advantage lies in reduced corrosion compared to other methods, thanks to the low water content in the vapor. However, the effectiveness of this method critically depends on ensuring that the instruments are completely dry before starting the process. Residual moisture can intensify corrosion due to the high reactivity of the chemical agents used, emphasizing the importance of precise control over pre-sterilization conditions [27]. Hidalgo et al. mention that the relevant biological indicators for this type of equipment are the endospores *G. stearothermophilus* and *Bacillus subtilis*, which they used in their study and obtained a satisfactory result, achieving 100% sterility under the conditions in which said equipment works [35].

Glutaraldehyde

Glutaraldehyde is one of the most widely used compounds in dental clinics and laboratories due to its high antimicrobial efficacy. Its use is particularly relevant in procedures involving direct or indirect contact with equipment, tools, or materials prone to contamination, such as dental impressions, where the risk of cross-infection is significant [34]. Fadaei reported in his study that glutaraldehyde concentrations ranging from 0.5% to 2% effectively inactivate coronaviruses on dental impressions. This is especially relevant in the context of viral pandemics, as it enables the implementation of preventive measures in medical and dental care settings [33].

It is important to note that, although glutaraldehyde is considered an effective disinfectant in dental settings, its use must be carefully controlled. Improper handling of concentrations and exposure times can pose health risks. Nevertheless, its application should be part of a broader infection control approach, incorporating both chemical and physical methods to minimize the risks of transmitting infectious diseases in these high-exposure environments [33,34].

In 2023, Salgar mentions that *Escherichia coli,* *Bacillus atrophaeus*, and *Clostridium difficile* are used to evaluate the efficiency of chemical sterilization processes such as glutaraldehyde. He mentions that this application is mainly used in healthcare environments and pharmaceutical laboratories. In addition, one of the advantages of using these microorganisms is that it allows continuous or real-time monitoring, thus avoiding contamination [36].

Hydrogen Peroxide

Hydrogen peroxide sterilization has emerged as a highly effective method for maintaining biosafety in dental clinics. This procedure significantly reduces bacterial loads in the clinical environment, thereby decreasing the likelihood of cross-infections. Its mechanism of action is based on oxidative stress inflicted on bacterial cells, achieved through the application of peroxide vapor during a cycle of approximately 20 minutes, with three minutes dedicated to application and 17 minutes to its chemotoxic effect. One of its key advantages is its ability to penetrate intricate surfaces and hard-to-reach areas, ensuring thorough disinfection. Additionally, this method leaves no toxic residues, as hydrogen peroxide decomposes into water and oxygen after the sterilization process. However, it is important to note that some sensitive materials may degrade with repeated exposure to this agent. Careful evaluation and specific protective measures are required before application to prevent damage. Even with these considerations, hydrogen peroxide remains an effective option for enhancing biosafety in dental practice environments [37].

Even so, the use of hydrogen peroxide is an effective option to improve the biosecurity of the spaces in which dental offices are located, which should be verified in the same way as other types of sterilization with biological indicators, the biological indicator of choice according to the literature for hydrogen peroxide are the endospores of *G. stearothermophilus* [37,38]. Fu et al. compared hydrogen peroxide vapor (HPV) and aerosolized hydrogen peroxide (aHP) for room disinfection using *S. aureus*, *C. difficile*, and *Acinetobacter baumannii*. HPV achieved a 6-log microbial reduction, outperforming aHP, which reached less than 4-log and showed uneven distribution. HPV also had safer leakage levels, making it more effective and reliable overall [39].

Radiation Sterilization

When discussing radiation sterilization, both UV and gamma rays are noteworthy methods, as they significantly affect the biological activities of microorganisms. Depending on the wavelength, the effects of radiation on biological materials can vary considerably, ranging from damage to the materials surrounding the microorganism to direct harm to the microorganism itself [31].

UV light: UV light is electromagnetic radiation with wavelengths ranging from 400 nm to 100 nm, divided into several segments: UVC (220-290 nm), UVB (290-320 nm), and UVA (320-400 nm). For sterilization procedures, the most commonly used wavelength is UVC, which is optimal for deactivating the DNA of pathogenic microorganisms. This radiation damages the nucleus, preventing replication and rendering the microorganisms non-infectious. In dentistry, UV light offers significant advantages. As noted by Ardeshna et al. in 2022, UV light does not alter material properties, is cost-effective, and is relatively easy to use [31]. For these reasons, it is suggested as a viable option for disinfecting orthodontic appliances before placement in the oral cavity. However, UV light has limitations. It cannot penetrate cracks or shaded areas, reducing its effectiveness in hard-to-reach spaces. Additionally, it can cause skin and eye damage, necessitating proper protective equipment during use. UV light can also damage or discolor certain materials, particularly plastics. Moreover, sterilization cycles may take anywhere from minutes to several hours, depending on surface reflectivity and the microorganisms targeted for elimination [26,31].

*Bacillus pumilus* is used as a biological indicator for the validation of UV light sterilization systems. Its spores, resistant to UV radiation, allow the effectiveness of exposure to this technology to be evaluated. The survival of the spores after the procedure indicates that the UV light system has not managed to adequately eliminate the microorganisms, thus providing a reliable tool to ensure the effectiveness of sterilization [40]. Siwe et al. evaluated the ZAPARAY™ UVC LED chamber (Eledricity Bvba, Merelbeke, Belgium) for disinfecting medical instruments contaminated with *S. aureus* ATCC 25923. UVC exposure achieved up to a 9-log₁₀ reduction on Petri dishes, 3.23-6.25 log₁₀ on non-rinsed dental tools, and varying reductions on other instruments. Rinsing before UVC exposure improved efficacy. The study suggests UVC chambers as a time-efficient disinfection alternative, but validation is required for specific instruments [41].

Gamma rays: Gamma rays are a form of radiation derived from the radioactive decay of atomic nuclei, making them widely used for sterilizing medical and dental devices and grafts. Due to their short wavelengths, gamma rays have high penetration power and energy, allowing them to effectively destroy microorganisms. Among their advantages, gamma rays can penetrate dense materials, making them effective for sterilizing complex medical devices. They do not easily react with materials, minimizing potential alterations and generating minimal residue, which reduces the need for additional cleaning procedures. Furthermore, gamma rays do not significantly heat the sterilized products, which is crucial for preserving heat-sensitive materials. However, gamma radiation has certain disadvantages. It can damage biomaterials used in medical devices, altering their composition and properties. Additionally, the radiation dose must be carefully controlled, as improper doses may cause undesired changes in materials. In conclusion, gamma radiation is a reliable and effective sterilization method, provided that radiation doses are meticulously managed [26,42]. The biological indicator of choice for gamma radiation sterilization is *B. pumilus*, due to the high resistance of its spores to ionizing radiation. McEvoy et al. compared gamma rays, X-rays, and electron beams (e-beam) for sterilizing medical devices using *B. pumilus* spores. All methods were equally effective at killing bacteria, working in a similar way across a dose range of 1-6 kGy, regardless of the dose rate [43].

Gas Sterilization

In dental offices, gas sterilization primarily involves the use of ethylene oxide gas and ozone.

Ethylene oxide gas: Ethylene oxide gas sterilization is used when heat causes unacceptable damage to instruments. This gas is an excellent sterilizing agent due to its high penetration capacity at room temperature. The sterilization process consists of five stages: preconditioning and humidification, gas introduction, exposure, evacuation, and air washing. The average cycle lasts approximately 2.5 hours, excluding the time required for residual gas removal. The main advantage of ethylene oxide gas is its effectiveness in sterilizing a wide variety of materials without causing damage, including plastic and electronic devices. However, it is a highly toxic and explosive reagent, documented as carcinogenic and associated with spontaneous abortions. Consequently, its use requires strict environmental controls, which result in high costs, making it less feasible for a conventional dental clinic [31,44].

In this type of sterilization, the biological indicator of choice is *B. atrophaeus*, due to the remarkable resistance of its spores to gas [38]. de Sousa Iwamoto et al. evaluated the effects of four sterilization methods, dry heat, autoclave, ethylene oxide, and gamma radiation, on the extracellular matrix (ECM) of dental scaffolds used for tissue regeneration [45]. All methods achieved complete sterilization while preserving the ECM molecular structure. Gamma radiation and ethylene oxide provided excellent results but were limited by high costs and accessibility issues.

Ozone: Ozone is a gaseous molecule with antimicrobial and antiviral properties, widely used for sterilizing environments such as dental offices. Its ease of penetration into porous and smooth surfaces allows it to effectively eliminate microorganisms through oxidation, destroying lipid structures, proteins, and nucleic acids. This makes ozone a promising option for sterilizing clinical environments, as it leaves no toxic residues, does not require additional products for activation, and can reach hard-to-access areas. However, it is important to note that high ozone concentrations can be toxic to humans, causing damage to the respiratory tract and mucous membranes. Therefore, strict precautions are necessary during its use, including proper ventilation and ensuring the absence of people in the room during the sterilization process [26]. De Souza Botelho-Almeida's study demonstrates that the use of *G. stearothermophilus* spores as a biological indicator shows evidence of the ability of ozone to exert an effective sterilizing effect [46].

Advanced technologies for decontamination

Recent advancements in decontamination technologies offer significant improvements for dental practices. HPV systems effectively disinfect hard-to-reach areas and are gaining traction in clinical settings for their ability to eliminate persistent contaminants. Ahmed and Mulder conducted a systematic review to evaluate the efficacy of HPV as a non-contact decontamination system for pathogens in dental environments [47]. Their study found that HPV significantly reduced microbial contamination, including bacteria, viruses, and spores, on surfaces commonly found in dental clinics. The authors highlighted its effectiveness in achieving high-level decontamination while being safe for sensitive dental equipment. Furthermore, HPV was shown to be a practical option for reducing infection risks in dental settings, particularly in high-touch areas and storage spaces, supporting its use as a supplementary method alongside traditional cleaning protocols.

UVC irradiation provides a chemical-free method to reduce microbial contamination on high-touch surfaces in dental operatories. Eslami et al. conducted a systematic review exploring the efficacy, safety, and applications of UV radiation in dentistry [48]. They reviewed 35 articles, highlighting that UV radiation effectively disinfects dental tools, surfaces, and materials, achieving significant reductions in microbial contamination, including bacteria, viruses, and fungi. Applications included disinfection of dental impressions, toothbrushes, prostheses, and titanium implants, with UVC radiation demonstrating the highest efficacy. However, the authors emphasized the importance of proper safety measures to minimize risks to skin and eyes from UV exposure. Querido et al. emphasized the importance of addressing contamination on surfaces, particularly in critical healthcare settings, and their findings are highly relevant to dentistry, especially regarding the disinfection of storage areas for sterilized instruments [49]. Dental clinics face significant challenges in maintaining sterility in storage spaces due to microbial contamination from environmental exposure and inadequate cleaning protocols. These areas are critical, as they directly impact the sterility of instruments used in patient care. The authors reviewed advanced self-disinfecting technologies, including antimicrobial coatings made of silver and copper, as well as photo-activated surfaces using titanium dioxide (TiO2), which demonstrated significant efficacy in reducing microbial loads on high-touch and storage surfaces. These technologies offer a promising solution for dental storage areas, where traditional manual cleaning and disinfection often fall short in addressing biofilm formation and residual contamination. Querido et al. also stressed the need for standardized protocols to evaluate the effectiveness of these materials in real-world settings, including dental practices [49]. By incorporating self-disinfecting surfaces in storage cabinets, instrument trays, and shelving, dental clinics can better ensure that sterilized instruments remain free from contamination until their next use. This innovation aligns with the broader goals of improving infection control and patient safety while reducing the reliance on manual cleaning methods, which can be labor-intensive and less effective. The implementation of such technologies in dental storage areas represents a critical step toward mitigating infection risks and enhancing overall clinical hygiene standards.

Fann et al. demonstrated the efficacy of far-infrared radiation (FIR) in preventing microbial contamination on the outer packaging of sterilized surgical instruments during storage [50]. FIR treatment effectively inhibited microbial growth, reducing colony-forming units (CFUs) to zero over a 30-day storage period, compared to 68.2% contamination in untreated packages, which included 34 bacterial species like *Staphylococcus *and *Bacillus* spp. FIR's ability to lower humidity in storage areas was identified as a key factor, creating an environment less conducive to microbial survival. In dental clinics, integrating FIR technology into storage areas for sterilized instruments can maintain sterility, reduce cross-contamination risks, and extend the shelf life of sterilized packages [50].

A study reviewed air quality management in dental settings, for example, in areas where sterilized instruments are stored [51]. Inadequate air quality in these storage areas can result in the recontamination of sterilized instruments through aerosols, microdroplets, and particulate matter (PM2.5 and PM10). Advanced air purification systems, such as high-efficiency particulate air (HEPA) filters and UVC radiation, were identified as highly effective in reducing airborne pathogens and maintaining sterility. The review highlighted the necessity of maintaining low levels of volatile organic compounds (VOCs) and ensuring continuous air circulation to minimize contamination risks. Regular maintenance of air purification systems, combined with natural ventilation, was also recommended to enhance effectiveness. Integrating air quality management into routine infection control practices is essential for ensuring that sterilized instruments remain uncontaminated and safe for clinical use, reinforcing the reliability of sterilization protocols in dental clinics [51].

Maintenance of autoclaves and dry heat sterilizers

Proper maintenance of sterilization equipment is one of the crucial factors in ensuring the efficacy of sterilization processes and the safety of patients in dental clinics. A sterilizer that undergoes continuous maintenance ensures that the processes performed are efficient and safe, minimizing the risk of cross-contamination or equipment failures. Regular maintenance also extends the equipment's lifespan, reducing operational costs associated with unplanned repairs and replacements. It is essential to emphasize that the maintenance of each sterilization device must be carried out strictly according to the manufacturer’s recommendations. These guidelines are designed to optimize the device's performance and ensure that operating conditions remain within optimal parameters. Following these instructions guarantees the reliability and effectiveness of the sterilization processes [52,53].

Autoclaves and dry heat sterilizers require specific maintenance procedures to ensure their optimal functioning and the effectiveness of sterilization cycles. Each type of equipment has unique maintenance requirements that must be rigorously followed according to established regulations. For autoclaves, the sterilization process involves placing water in the reservoir chamber. Manufacturers recommend avoiding tap water, as it contains dissolved minerals that can lead to scale buildup in the heater and chamber, causing malfunctions. Instead, distilled or double-distilled water is recommended for use in autoclaves. However, one disadvantage of this type of water is that, despite its low mineral content, the exact mineral quantity is unknown, and it may still be contaminated with endotoxins or pathogens. Therefore, it is crucial to ensure that the autoclave completes the sterilization cycle correctly to maintain its effectiveness and reliability [52].

Guidelines for infection prevention and control in dentistry

Pankhurst and Coulter, in their book "Basic Guide to Infection Prevention and Control in Dentistry" highlight five essential points for autoclave care [52]. First, it is important to use only distilled water and avoid using tap water, as tap water contains minerals that can damage the equipment. Second, the equipment should be periodically disinfected following the manufacturer's instructions. Third, any water discharged by the equipment should be disposed of and not stored to avoid contamination. Fourth, at the end of each day, the reservoir should be cleaned using a lint-free disposable cloth. Finally, the reservoir should be left empty until the next use.

Regarding dry heat sterilizers, since distilled water is not required, some of the previously mentioned instructions do not apply. However, it is essential to inspect the seals or gaskets, replace filters, and lubricate the hinges. These maintenance tasks should also be performed on autoclaves. Additionally, dry heat sterilizers require regular inspections to ensure their proper functioning [53]. Sterilization equipment, whether autoclaves or dry heat sterilizers, should undergo a complete maintenance check at least once a year. This includes calibration by a qualified expert to ensure the equipment complies with established standards and functions optimally. Regular verification of sterilization equipment is critical to maintaining its effectiveness and ensuring safety in dental practices [53].

Checks of dry heat and autoclave equipment

It is crucial that sterilizers receive periodic performance checks to ensure the effectiveness and safety of their sterilization processes. Safety and maintenance tests of the equipment must be carried out at least once a year, while users must perform daily, weekly, and monthly checks, as well as keep a record of these tests, which can be carried out in different ways. Annual or semi-annual inspections must be carried out by trained engineers to check the safety systems and calibrate the equipment controls. In addition, monitoring temperature and pressure in each cycle, along with the use of physical, chemical, and biological indicators, is essential to ensure adequate infection control and quality in sterilization processes [25].

Verification With Physical Indicators

Physical indicators refer to the devices incorporated in sterilization equipment such as thermometers, pressure gauges, and timers in autoclaves, and thermometers and timers in dry heat ovens. Each equipment has detailed specifications in its operating manuals which must be followed to ensure the correct operation of these instruments. Constant monitoring of these indicators allows for the detection of equipment failures and the taking of corrective measures in a timely manner. However, it is important to mention that although physical controls function properly, their correct operation does not necessarily guarantee that the sterilization process is being carried out effectively, but only that the parameters mentioned by these indicators are being reached [54].

Verification With Chemical Indicators

Chemical monitoring of sterilization cycles uses heat-sensitive indicators that change color when exposed to different temperatures. These products are commercially available in various forms, for example, impregnated in paper strips, in adhesive tape used to package instruments (witness tape), or impregnated in the prefabricated bags used to wrap surgical instruments. However, these indicators only reflect the conditions in the area where they are located, so it is advisable to use several in different areas of the equipment. Some of these indicators are not temperature-specific, and therefore will only indicate their exposure to steam but not the specific temperature at which the equipment reached, so they must be combined with other methods to ensure the effectiveness of the process. However, it is a useful indicator for the user, since it ensures that the instrument went through the sterilization cycle, although it does not ensure the quality and complete bacterial death in said process [25].

Verification With Biological Indicators

In preventing cross-infections in dentistry, the American Dental Association (ADA), Organization for Safety, Asepsis and Prevention (OSAP), and Centers for Disease Control and Prevention (CDC) recommend weekly verification of sterilization cycles using biological indicators, a standard in the United States. In addition, WHO notes that biological controls are currently the only method to confirm the sterilization of articles and evaluate the effectiveness of the process. WHO also recommends weekly verification of autoclaves and dry heat, adjusting the frequency based on equipment use and after repairs or when sterilizing prostheses or implants [55,56].

As established by NOM-013-SSA-2015, the sterilization process is fallible, so it is essential to continuously monitor the equipment using biological indicators. These indicators are the only tools accepted worldwide to evaluate the effectiveness of sterilization processes. In the context of Mexico, the standard establishes specific guidelines to reduce the risk of disease transmission between the patient and the dentist. Among the most relevant measures are the disinfection and sterilization of instruments, as well as the obligation to carry out bi-monthly monitoring using biological indicators to control the sterilization cycles of the equipment used by dental surgeons. As stipulated in section 8.24 of the standard, dentists must "Apply biological witnesses every two months, as quality control of the sterilization cycles and keep a record of the results," in compliance with the provisions of the Pharmacopoeia of the United Mexican States. This provision has become mandatory since its publication in the Official Gazette of the Federation [29]. As stated in NOM 013, the sterilization process is fallible, making it essential to continuously monitor equipment using biological indicators. These indicators are the only globally accepted tools for evaluating the effectiveness of sterilization processes [29].

Verification With Biological Indicators

Specifically, to evaluate the sterilization process of autoclaves and dry heat sterilizers, the endospores of *G. stearothermophilus* are used for autoclaves, while *B. atrophaeus* is used for dry heat processes. These endospores provide an objective confirmation of whether sterilization equipment is functioning correctly, as they assess the elimination of highly resistant bacterial spores. Regular use of these indicators is vital to ensure that sterilization protocols are properly followed, thereby contributing to patient safety [32].

Use of biological indicators in sterilization processes

The use of biological indicators in sterilization processes involves placing these indicators inside the equipment before starting the sterilization cycle. The cycle is then executed according to the previously established parameters. Once the process is complete, the indicators are processed to evaluate their effectiveness. There are two possible outcomes when assessing the biological indicators. A result with no bacterial growth indicates that the sterilization equipment has functioned correctly. Conversely, the presence of bacterial growth suggests that the sterilization procedure was ineffective. This is critical, as a sterilization process is defined by the complete elimination of pathogenic agents [32,52].

Addressing bacterial growth results in sterilization processes

When bacterial growth is detected during sterilization validation, it is essential to conduct a thorough inspection of the sterilization equipment to identify potential failures and issues. This ensures that the instruments used in dental care are fully sterile and safe for patient use. Using biological indicators, authors such as Patiño-Marín et al. [57] and Chanchareonsook et al. [58] have provided a list of the most common errors encountered during sterilization processes.

Patiño-Marín et al. observed that errors made by the personnel in charge of carrying out the sterilization process and mechanical failures in the sterilization cycles are made evident through the use of biological indicators [30]. According to the analysis carried out in their study, it was identified that the person in charge of the sterilization process carried out the process with inadequate temperatures, times, and preheating, which compromised the effectiveness of the cycle and put the quality of the sterilization process at risk.

In another study conducted by Patiño et al. in 2015, several flaws in the sterilization process were identified [57]. Among the most common errors were deficiencies in the procedure, such as incorrect regulation of temperature, time, or pressure during the sterilization cycle. In addition, the lack of adequate supervision of the procedure by the personnel in charge was highlighted, as well as insufficient maintenance of the equipment, which contributed to compromising the effectiveness of the sterilization processes. In 2022, Chanchareonsook et al. determined that the failures in the sterilization procedure in their investigation were caused by human error [58]. Their investigations revealed that the incident occurred because the on-duty staff failed to properly activate the steam sterilizer, which prevented the sterilization process from being carried out properly, thus compromising the effectiveness of the cycle and the safety of the sterilized equipment.

In 2024, Patiño-Marín et al. identified several factors that contribute to bacterial growth in sterilization cycles [32]. Among the main causes of sterilization failures, they highlighted the lack of staff training, inadequate equipment maintenance, incorrect execution of the procedure, and poor interpretation of biological indicators. Their study emphasizes that insufficient training and inappropriate use of equipment are critical factors that compromise the effectiveness of sterilization, underlining the importance of adequate training and correct maintenance and operation practices to ensure infection control. It is important to emphasize that the main factor that affects the failure of sterilization cycles is the lack of knowledge of the personnel regarding the appropriate times and temperatures for each process. Although proper maintenance of the equipment and correct execution of the procedure are relevant factors, insufficient training in these critical aspects significantly compromises the effectiveness of sterilization, which highlights the need for comprehensive training and constant updating in sterilization practices.

Both authors highlight that bacterial growth in sterilization cycles often results from human error or equipment malfunction. Therefore, dental clinics and offices must implement comprehensive operational plans to ensure these procedures are performed correctly and consistently, maintaining high standards of safety and effectiveness [32,58].

Storage of sterilized dental instruments

Proper storage of sterilized dental instruments is crucial to maintaining their sterility and ensuring patient safety in dental practices. Inadequate storage practices can compromise the sterility of instruments, leading to HAIs and potential legal liabilities for dental professionals [59].

In dental settings, the storage of sterilized instruments must comply with strict guidelines to maintain sterility. Sterile storage areas should be clean, well-organized, and designed to prevent contamination from environmental factors such as dust, moisture, and microorganisms. Proper ventilation and temperature control are essential to prevent the degradation of packaging materials. For example, the CDC recommends storage conditions of 20-25°C and 30-60% relative humidity [60]. In dental clinics, storage areas should be separate from patient treatment areas to minimize the risk of cross-contamination. Designated storage zones for clean, sterilized, and used instruments enhance workflow efficiency and safety. Clear labeling and color-coded systems can help staff quickly identify and handle instruments appropriately [61,62].

Bruna et al. investigated the effects of environmental temperature and humidity on the sterility of surgical instruments stored in various packaging materials [63]. Their study demonstrated that instruments stored under high-temperature (32.8°C) and high-humidity (75.8%) conditions, as well as standard conditions (21.6°C and 68.4% humidity), maintained sterility for 30 days when packaged in woven textiles, nonwoven Spunbond Meltblown Spunbond (SMS), crepe paper, or plastic pouches. Despite deliberate external contamination, no microbial growth was observed, emphasizing the effectiveness of high-quality packaging in maintaining sterility. However, the authors cautioned that improper handling and storage could compromise sterility. These findings highlight the importance of using robust packaging and adhering to standardized storage protocols in dental clinics to ensure the safety and sterility of instruments [63].

Sterile storage must also consider the specific needs of dental materials, such as high-speed handpieces and endodontic files, which can be more sensitive to environmental factors. For instance, high-speed handpieces require storage in conditions with controlled humidity, as excessive moisture can lead to internal corrosion and damage to bearings, compromising their performance [29]. Endodontic files, due to their fine structure, must be stored in rigid containers or specialized trays to prevent deformation. Packaging materials for these items should include moisture-resistant barriers to safeguard against environmental fluctuations. Routine inspections should be conducted to ensure that packaging remains intact and instruments are free from contamination [59]. These measures are essential to maintain the functionality and sterility of these critical tools. Training staff on these specific requirements is critical to maintaining optimal conditions.

Compatibility between sterilization and labeling processes

Materials and packaging systems used for sterilized dental instruments must be compatible with the sterilization process. ISO 11607-1 (2006) outlines that the material's performance must remain within specified limits even after exposure to sterilization processes such as steam, ethylene oxide, or gamma irradiation. Compatibility ensures that the sterilization process does not degrade the packaging or affect the sterility of its contents. Packaging systems must also be designed to accommodate labeling systems that remain intact and legible until the point of use [64].

Proper labeling of sterilization bags is critical for maintaining traceability and ensuring effective sterilization workflows in dental and medical settings. Labels should not react with the materials or impair the utility of the sterile barrier system. The markers used for this purpose should be resistant to the specific sterilization processes used, including steam, ethylene oxide, and hydrogen peroxide plasma, as these methods expose materials to high temperatures, pressure, and chemicals that can degrade ordinary ink [65]. Additionally, the markers must not damage or weaken the material of the sterilization bags, as this could lead to contamination. Specialized markers like VWR Lab Markers (VWR, Inc., West Chester, Pennsylvania, United States) and Fisherbrand Sterilization Markers (Thermo Fisher Scientific Inc., Waltham, Massachusetts, United States) are designed to withstand sterilization conditions, ensuring clear, durable markings that do not compromise packaging integrity. The use of industrial-grade markers, such as Sharpie Industrial (Newell Brands Inc., Atlanta, Georgia, United States) may also be appropriate for certain sterilization environments, provided they meet compatibility standards. Modern labeling systems may also incorporate barcodes or radio-frequency identification (RFID) technology, allowing for automated tracking of instruments through sterilization, storage, and usage cycles. This integration improves workflow efficiency and reduces human error, making it a valuable addition to high-volume dental and medical practices [66].

Sterile storage and transport of dental instruments

Correct packaging of instruments provides a barrier against microorganisms [67]. The maximum storage duration for sterilized dental instruments depends on several factors, including the type of packaging material, storage conditions, and the sterility assurance level (SAL) established during the sterilization process. Event-related sterility is now widely accepted over time-related sterility, meaning instruments remain sterile unless compromised by an event such as package damage, exposure to moisture, or handling errors. However, best practices still provide general guidelines for storage times: Instruments stored in single-use pouches and wraps, typically made of paper/plastic sterilization material, can be stored for up to six months in controlled environments with proper handling. Sterilized instruments stored in validated rigid containers with intact seals may remain sterile for up to one year if storage conditions are maintained. On the other hand, reusable cloth wraps, which are less durable compared to paper/plastic pouches, are generally recommended for shorter storage durations, usually no more than one month [60].

Some studies highlight the effectiveness of various sterilization packaging materials in maintaining sterility over extended periods. Chang et al. found that crepe paper and nonwoven wraps preserved sterility for up to six months, while paper/plastic pouches lasted up to nine months under controlled conditions [68]. Similarly, Lakhan et al. demonstrated that materials like kraft paper and laminated pouches could maintain sterility for up to 24 months under proper handling and environmental control, endorsing event-based sterility models [69]. Puangsa-Ard et al. confirmed the durability of paper/plastic pouches after multiple sterilization cycles and six months of storage, provided their integrity was preserved. Additionally, a study comparing tape-sealed paper envelopes, heat-sealed nylon tubing, and paper/plastic peel pouches found all three materials effective for up to one year, with paper/plastic peel pouches showing 0% contamination due to their superior barrier properties [70]. Another comparative study evaluated the sterility of instruments stored under different conditions in a clinical setting, and it was found that instruments stored in backpacks without previously disinfected plastic containers had higher contamination levels compared to those stored in disinfected plastic boxes [71]. In the study by Dreikausen et al., no microbial load could be found on sterile tools stored in reusable containers in the sterile storage area [72]. These findings underscore the importance of using appropriate, disinfected containers for storing sterilized instruments.

According to ISO 11607-1, the storage and transport of sterilized dental instruments must protect against environmental factors that could compromise sterility. Key considerations include maintaining appropriate temperature and humidity levels, ensuring the physical integrity of the sterile barrier system, and using protective outer packaging when necessary. Storage areas should facilitate aseptic presentation and prevent mechanical damage to the packaging. During transportation, the use of rigid containers with secure closures can minimize the risk of punctures or tears in the sterile barrier system. Furthermore, transport carts used within dental clinics should be dedicated to sterile items and regularly disinfected to maintain cleanliness [64].

Design and development requirements for packaging systems

General Requirements

The design of packaging systems should minimize safety hazards under specified conditions of use. Packaging must provide physical protection and maintain the sterile barrier system's integrity throughout storage and handling. This ensures that sterility is maintained until the point of use or the expiration date [64].

Design Elements

The design process should consider factors such as the weight and configuration of dental instruments, labeling requirements, environmental conditions, and sensitivity to mechanical shocks or static discharge. For dental instruments like endodontic files and mirrors, the sterile barrier system must also support aseptic presentation to the user. Innovative designs, such as peelable pouches with clear visibility of the instrument inside, can improve usability and reduce the risk of contamination during handling. Additionally, the use of multi-layer materials with enhanced puncture resistance can extend the shelf life of packaged instruments [64].

Performance and stability testing

Performance testing ensures that the sterile barrier system maintains integrity after exposure to sterilization and handling processes. Real-time aging tests are essential to demonstrate the system's ability to maintain sterility over its intended lifespan. Accelerated aging tests may be used as interim evidence for expiry dates until real-time data becomes available [64]. Stability testing evaluates how the packaging materials respond to varying storage conditions, such as fluctuations in temperature and humidity. This testing is particularly relevant for dental clinics in regions with extreme climates [64].

Packaging integrity and sterility maintenance

In dentistry, maintaining packaging integrity is essential for preserving the sterility of instruments such as dental handpieces, burs, and mirrors. Sterilized items must be stored in their original packaging until use, as damaged packaging can result in contamination. The integrity of packaging materials, such as sterilization wraps, pouches, and containers, must be regularly inspected. Studies have shown that proper packaging significantly reduces the risk of contamination during storage [60].

Reusable sterilization packaging systems, such as rigid containers and woven wraps, require rigorous inspection before reuse to prevent breaches in sterility. The Association of periOperative Registered Nurses (AORN) guidelines emphasize the importance of verifying packaging integrity through lighted inspection tables and following manufacturer recommendations for reuse cycles [62]. Additionally, Klumdeth et al. demonstrated that reused paper/plastic sterilization pouches, when carefully inspected and monitored, can maintain sterility, offering a cost-effective solution for dental clinics [61].

Reuse of Sterilization Pouches

The reuse of sterilization pouches has been explored as a cost-effective measure in dental practices, particularly in settings where resources are limited. Studies indicate that paper/plastic sterilization pouches can be reused up to three times without compromising sterility, provided that stringent inspection protocols are followed. Reused pouches must be inspected for signs of wear, such as tears, seal degradation, or loss of integrity, which can lead to contamination risks [61]. To ensure safe reuse, it is recommended that pouches undergo physical and chemical indicator tests after each cycle to confirm their continued effectiveness. However, the adoption of reusable pouches requires staff training and adherence to detailed guidelines to prevent errors that could compromise sterility. While this approach offers economic benefits, the decision to reuse pouches must be balanced with potential risks and the need for meticulous quality control [61,67].

Fernandes demonstrated that paper/plastic sterilization sleeves can be reused without compromising sterility or integrity, even after 153 days of storage in an open environment [73]. While the experimental group and the negative control showed no signs of contamination, the positive control group, intentionally contaminated, exhibited a high number of colony-forming units. These findings highlight the feasibility of reducing plastic waste in dentistry through the controlled reuse of sterilization sleeves, provided strict handling and storage conditions are maintained. The author emphasizes the need for further research to establish reuse limits and evaluate the applicability of this practice in real clinical settings, thereby promoting more sustainable dental care. Rizan et al. analyzed the environmental and financial impact of decontaminating and packaging reusable surgical instruments, finding that individually packaged items have a significantly higher carbon footprint (189 g CO2e per instrument) compared to those in sets (66-77 g CO2e per instrument) [74]. Strategies such as efficient machine loading, using renewable energy, and integrating rarely used instruments into existing sets effectively reduced costs and environmental impact. These findings are relevant to dentistry, where reusable instruments are routinely sterilized and packaged. Adopting sustainable practices like maximizing sterilization loads and using eco-friendly packaging materials can help dental practices reduce their carbon footprint while maintaining safety and sterility, aligning with global efforts to minimize healthcare's environmental impact [74].

## Conclusions

Effective sterilization in dental practices requires strict adherence to key steps to ensure patient safety and infection control. The process begins with thorough pre-cleaning and cleaning of instruments to remove organic and inorganic residues, followed by appropriate disinfection for non-critical, semi-critical, and critical items. Instruments must be completely dried to prevent corrosion before sterilization, which is performed using methods like steam, dry heat, chemical sterilization, or radiation, depending on the material's properties. Verification with biological indicators is essential to confirm sterilization effectiveness, and proper storage protocols, such as sealed containers or pouches, help maintain sterility until use. Adhering to these steps ensures reliable sterilization, reduces contamination risks, and promotes safe clinical practices. Advances in technology offer new opportunities to enhance sterile storage practices, but the commitment to basic principles remains paramount. Addressing challenges and embracing future innovations will require a collaborative effort among stakeholders.
